# *Salmonella* in Wild Birds Utilizing Protected and Human Impacted Habitats, Uganda

**DOI:** 10.1007/s10393-016-1149-1

**Published:** 2016-08-03

**Authors:** Josephine Azikuru Afema, William M. Sischo

**Affiliations:** Department of Veterinary Clinical Sciences, College of Veterinary Medicine, Washington State University, P.O. Box 647010, Pullman, WA 99164 USA

**Keywords:** anthropogenic activities, environmental contamination, *Salmonella* transmission, human–wildlife interface, wild birds, Uganda

## Abstract

As human populations in Africa expand, humans encroach and modify wildlife habitats for farming, fishing, tourism, or settlement. Anthropogenic activities in shared environments may promote transmission of zoonotic pathogens between humans, domestic animals, and wildlife. Between July 2012 and February 2014, we evaluated *Salmonella* prevalence, serovars, genotypes, and antibiotic resistant phenotypes in resident and migratory birds utilizing human-impacted habitats in northwestern Lake Victoria and protected habitats in Queen Elisabeth National Park. *Salmonella* occurrence in the urban environment was assessed by sampling storm-water and wastewater from a channel that drains Kampala City into Lake Victoria. *Salmonella* was detected in 4.3% pooled bird fecal samples, and 57.1% of environmental samples. While birds in impacted and protected areas shared serovars, the genotypes were distinct. We found distinct strains in birds and the environment suggesting some strains in birds are host adapted, and strains circulating in the environment may not necessarily disseminate to birds. Conversely, birds in both impacted and protected areas shared strains with the urban environment, suggesting *Salmonella* disseminates between impacted environments and birds across sites. Overall, more strains were observed in the urban environment compared to birds, and poses risk of *Salmonella* reemergence in birds and transmission across species and space.

## Introduction

Populations of some Afro-Palearctic migratory birds have been declining in Europe for decades (Sanderson et al. 2006). In Uganda, a decline in wild bird (hereafter bird) populations has been observed since 2004 (Pomeroy and Asasira 2011). While the exact cause of these declines is unclear, several interconnected factors may contribute including increasing human population resulting in habitat loss, increasing climatic variability affecting breeding and wintering grounds, and decreasing food availability in key habitats (Sanderson et al. 2006; Vickery et al. 2014). Another factor is the potential for pathogen introduction into bird populations when humans encroach and modify wildlife habitats into agricultural, fishing, or urban areas. Increasing habitat overlap may promote pathogen transmission between humans and wildlife (Daszak et al. 2000). Also, multiple migratory bird species from various breeding grounds congregate at higher densities at stopover and wintering grounds and intermingle with resident birds. Such phenomena may promote pathogen exposure and transmission across species and space (Altizer et al. 2011).

Zoonotic pathogens can threaten bird populations sharing habitats with humans. Non-typhoidal *Salmonella* affects humans worldwide (Majowicz et al. 2010) and multiple animal hosts, and causes morbidity and mortality in birds under natural and human influenced conditions (Pennycott et al. 1998; Velarde et al. 2012; Giovannini et al. 2013). For example, provisioning has been shown to attract high bird densities at feeding sites leading to fecal contamination, pathogen transmission, and reemergence of salmonellosis (Pennycott et al. 1998). *Salmonella* is also frequently isolated from apparently healthy scavenging birds that are attracted to refuse pits and human sewage outfalls (Benskin et al. 2009). Asymptomatic birds may disseminate *Salmonella* to susceptible individuals through fecal shedding, shared environments, and via direct contact.

Few studies have compared *Salmonella* prevalence across diverse bird species under non-epidemic conditions (Hernandez et al. 2003); urban and rural settings (Hamer et al. 2012); or different habitat protection types. These studies have mostly been conducted at European and North American sites (Pennycott et al. 2006; Hughes et al. 2008; Hamer et al. 2012). Studies at African sites are important because Afro-Palearctic migratory birds spend a considerable part of their annual cycle in Africa. For instance, Uganda has wetland habitats of international importance for resident and migratory birds (Byaruhanga and Nalwanga 2006). The bird habitats in northwestern Lake Victoria are closely associated with Kampala city, and evidence of microbial contamination in the urban environment has been documented (Afema et al. 2016). There are calls for research and conservation efforts to understand and mitigate factors causing Afro-Palearctic migrant bird population declines (Vickery et al. 2014).

This study investigated whether birds utilizing human-impacted habitats in northwestern Lake Victoria had higher risk of acquiring *Salmonella* compared to birds in Queen Elisabeth national park (QENP). The study used noninvasive methods to collect fecal samples from birds. *Salmonella* occurrence in the urban environment was assessed by sampling storm-water and wastewater from a channel that drains Kampala City into Lake Victoria. This study describes *Salmonella* occurrence, antibiotic-resistant phenotypes, serovar distribution and genotypic structure of common serovars, and infer transmission.

## Materials and Methods

### Study Area

**Figure 1:**
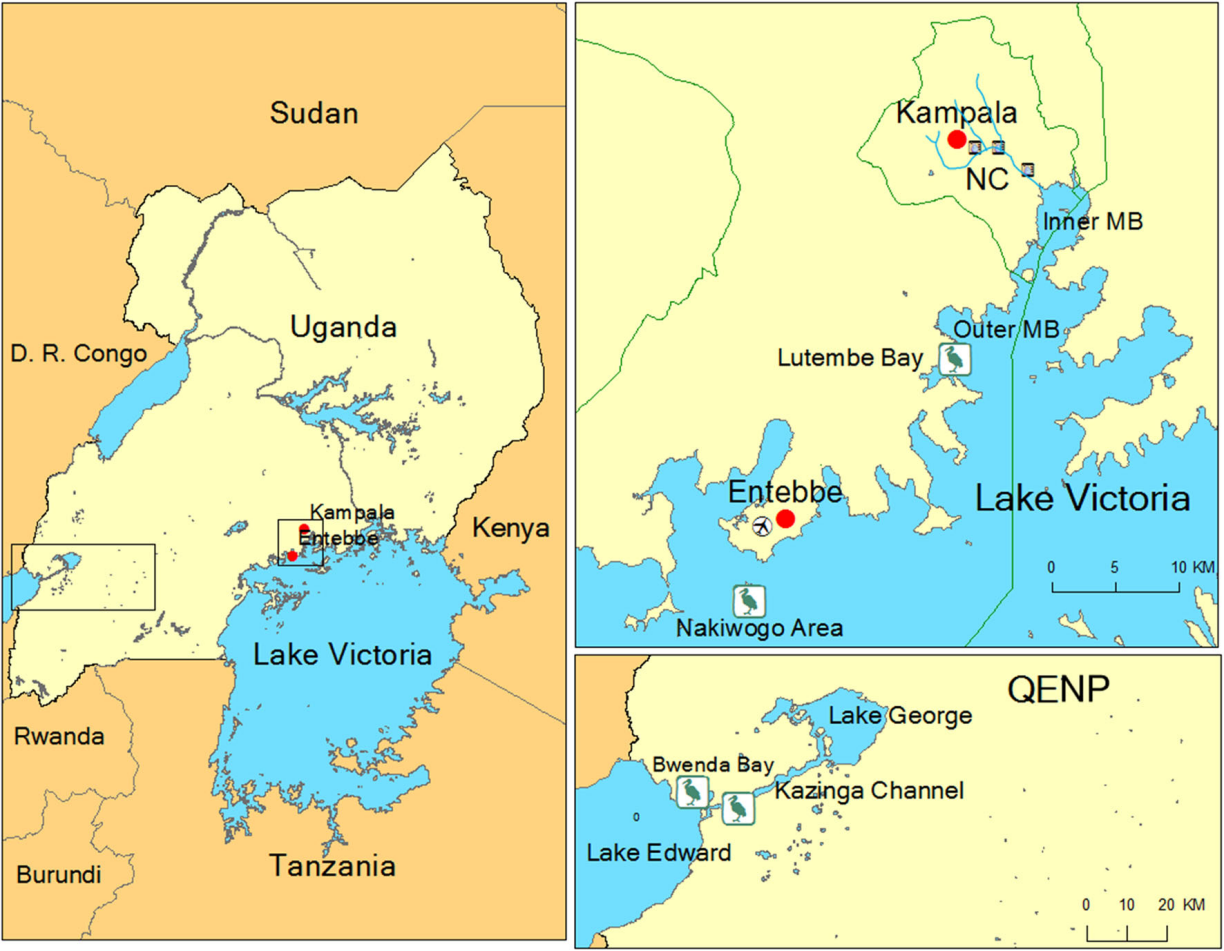
Map of Uganda showing study area. *Inset* on the *upper right* is northwestern Lake Victoria showing spatial relationship between Lutembe Bay, Nakiwogo, Nakivubo Channel (NC), Murchison Bay (MB), and the main cities. The Queen Elisabeth (QENP) sampling sites (Bwenda Bay and Kazinga Channel) and their spatial relationships are shown on the *lower right*. Maps obtained from: ArcGIS\Packages\0ef9b6b4700942ef9adaecfb0b3857cd\commondata\data0\Ug_Districtboundaries2006.shp. http://services2.arcgis.com/D6cgxWzepFC1quJH/arcgis/rest/services/Uganda_Rivers/FeatureServer. ArcGIS\Packages\2ac6a57b073749d4b2ab2ba90efabb55\commondata\data0\Ug_Waterbodies.shp.

This study was conducted in Lake Victoria region and in QENP (Fig. 1). Lutembe Bay and Nakiwogo are important bird areas in northwestern Lake Victoria (LVBA). Lutembe Bay is located on outer Murchison Bay and is spatially close to Kampala, the capital and largest city in Uganda. Lutembe Bay has many small muddy islets surrounded by shallow waters that birds use for roosting (Byaruhanga and Nalwanga 2006). Nakiwogo is located close to Entebbe town. It covers a greater geographic area and has less human impact compared to Lutembe Bay and birds roost on rocky outcrops in the lake and the shorelines. Fishing communities exist at both Lutembe Bay and Nakiwogo.

Nakivubo Channel (NC) is approximately 12-km-long channel network constructed to drain storm-water from Kampala City. This channel also receives secondary effluent from wastewater treatment plant, and abattoir run-off and it empties into inner Murchison Bay in Lake Victoria. Impairment of water quality because of high fecal coliform counts has been documented in NC and Murchison Bay (Kansiime and van Bruggen 2001).

QENP is a savanna park located in western Uganda. Human activity in the park is restricted to tourism, staff and permitted fishing villages. Bwenda Bay is located on Lake Edward close to Katwe town. The bay has shallow waters that are shielded from the lake by wetland plants. Kazinga Channel is a 33 km stretch of water that connects Lake George and Lake Edward. Kazinga Channel is popular for tourism and birds commonly roost on the southern shoreline. Fishing is permitted in some parts of the channel with a resident community at Kazinga fishing village.

### Sampling Strategy and Sample Collection

This study employed a repeated cross-sectional sampling approach. From July 2012 to February 2013, 1 l of grab water samples from 3 points along NC was collected into sterile plastic bottles. From December 2012 to February 2013, fecal samples were collected from birds at Lutembe Bay, Nakiwogo, Bwenda Bay and Kazinga Channel. Birds were identified during sampling or photographed and identified later using a guidebook (Stevenson and Fanshawe 2006). A cotton-tipped swab was used to collect a single fresh fecal dropping on roosting grounds, and 5 fecal swabs were pooled into a 50-ml tube containing 45 ml Rappaport–Vasilliadis selective enrichment broth (R10, Hardy Diagnostics, USA). At each site, samples were collected 1–3 times per month. From October 2013 to February 2014, samples were collected once a month from all sites. Samples were placed in a cool box containing cold packs and processed within 24 h of collection at the Microbiology Laboratory, College of Veterinary Medicine, Makerere University or a field laboratory in Mweya, QENP.

### Culture Methods

Samples in R10 were incubated at 42°C for 18–24 h, then 100 μl of R10 enriched broth was spread onto xylose lysine tergitol-4 (XLT-4) or xylose lysine deoxycholate (XLD) agar plates (Difco Laboratories) using sterile beads, and incubated at 37°C for 18–24 h. Based on selective plates indicating *Salmonella* growth, original enriched broths suspected to contain *Salmonella* were subjected to serial dilutions (Sanderson et al. 1995) and re-plated onto XLD or XLT-4 plates to obtain well-isolated colonies. An average of 9 suspect *Salmonella* colonies were picked from each plate and stabbed into brain heart infusion agar (Hardy Diagnostics, USA) banking tubes and stored at room temperature. Water samples from NC were processed using previously described methods (Berge et al. 2006) with minor modifications (Afema et al. 2016). All isolates were shipped to Washington State University (WSU) for further processing in compliance with Uganda National Council of Science and Technology materials transfer agreement with College of Veterinary Medicine, Makerere University.

### Serotyping

Suspect *Salmonella* isolates were screened by PCR amplification of the *inv*A gene (Yoshida et al. 2007). PCR-positive samples were then evaluated using a genovar method and assigned a genovar code (Leader et al. 2009). All isolates were subsequently subjected to the Kauffman–White serotyping scheme (Popoff et al. 2004).

### Multiple Locus Variable Number Tandem Repeats Analysis (MLVA)

*Salmonella* Typhimurium were genotyped using MLVA (Lindstedt et al. 2003, 2004) according to PulseNet, Centers for Disease Control and Prevention protocol. Briefly, DNA was prepared as boiled cell lysate and seven variable number tandem repeats (VNTR) loci (ST3, ST5, ST7, STTR10pl, ST2, ST6, and ST8) were amplified. The PCR products were separated by capillary electrophoresis at the WSU Laboratory for Biotechnology and Bioanalysis. The copy number (allele) for each VNTR locus was determined as follows: [(observed fragment size − offset)/repeat size]. Based on alleles at the 7 loci, isolates were assigned as a unique MLVA type. The data were analyzed using PHYLOViZ software (Francisco and Vaz 2012).

### Pulsed Field Gel Electrophoresis (PFGE)

We used PulseNet PFGE protocols (Ribot et al. 2006) to subtype non-*S*. Typhimurium serovars recovered from at least two sampling sites. DNA was cleaved with restriction endonuclease XbaI (Fermentas, Thermo Fisher Scientific). PFGE bands were assigned and analyzed using BioNumerics 6.6 software (Applied Maths, Austin, TX, USA). The Dice coefficient was used to assess band pattern similarity and isolates with ≥90% similarity index were considered to be closely related. Cluster analysis was performed by the unweighted pair group method with arithmetic mean, band matching tolerance of 2%, and relaxed doublet matching.

### Antibiotic Resistance Tests

Isolates were tested for resistance to a panel of 15 antibiotics (amikacin, 30 μg; amoxicillin/clavulanic acid, 20/10 μg; ampicillin, 10 μg; cefotaxime, 30 μg; cefoxitin, 30 μg; ceftiofur, 30 μg; chloramphenicol, 30 μg; ciprofloxacin, 5 μg; gentamicin, 10 μg; kanamycin, 30 μg; nalidixic acid, 10 μg; streptomycin, 10 μg; sulfisoxazole, 250 μg; tetracycline, 30 μg; and trimethoprim/sulfamethoxazole, 1.25/23.75 μg;) using a disk diffusion assay (Bauer et al. 1966). Isolates were classified as resistant or susceptible to each antibiotic using Clinical and Laboratory Standards Institute definitions for *Enterobacteriaceae* (Watts 2008) except for streptomycin where the National Antimicrobial Resistance Monitoring Systems breakpoint was used (CDC 2013). Isolates with intermediate resistance were interpreted as susceptible. A single resistance profile was created for each isolate by concatenating resistance to 15 antibiotics.

## Results

### Birds Observed During Sample Collection

The species observed during sample collection are shown in Table 1. We mostly observed mixed species flocks; therefore, the species from which a fecal sample was collected could not be ascertained. The species that were present at all sites during most sampling occasions include black-winged stilt, little egret, gull-billed tern, grey heron, little ringed plover, Egyptian geese, and grey-headed gull. African skimmers were observed only at Kazinga Channel during all sampling occasions. Pied avocet, lesser and greater flamingos were only observed at Bwenda Bay. The common Squacco heron, long-toed lapwing, saddle-billed stork, and black-tailed Godwit were only observed at Lutembe, while black kite, barn swallow, whiskered tern, and yellow wagtail were only observed at Nakiwogo area.

**Table 1:** List of Birds Observed During Sample Collection

Family	Scientific name	Common name	Migrant	Lutembe	Nakiwogo	Kazinga	Bwenda
Accipitridae	*Milvus migrans*	Black kite	Resident	No	Yes	No	No
Alcedinidae	*Ceryle rudis*	Pied kingfisher	Resident	Yes	Yes	Yes	No
Anatidae	*Alopochen aegyptiacus*	Egyptian geese	Resident	Yes	Yes	Yes	Yes
	*Anas undulata*	Yellow-billed duck	Resident	Yes	Yes	Yes	No
Ardeidae	*Ardea cinerea*	Grey heron	Resident	Yes	Yes	Yes	Yes
	*Ardea goliath*	Goliath heron	Resident	No	No	No	Yes
	*Ardea melanocephala*	Black-headed heron	Resident	Yes	Yes	No	No
	*Ardeola ralloides*	Common Squacco heron	Resident	Yes	No	No	No
	*Egretta garzetta*	Little egret	Resident	Yes	Yes	Yes	Yes
	*Mesophoyx intermedia*	Yellow-billed egret	Resident	Yes	Yes	No	No
Burhinidae	*Burhinus vermiculatus*	Water thicknee	Resident	No	Yes	Yes	No
Charadriidae	*Charadrius dubius*	Little ringed plover	Migrant	Yes	Yes	Yes	Yes
	*Charadrius pecuarius*	Kittlitz’s plover	Resident	Yes	Yes	Yes	No
	*Vanellus crassirostris*	Long-toed lapwing	Resident	Yes	No	No	No
	*Vanellus spinosus*	Spur-winged lapwing	Resident	No	Yes	Yes	Yes
Ciconiidae	*Anastomus lamelligerus*	Open-billed stork	Resident	Yes	No	No	Yes
	*Ephippiorhynchus senegalensis*	Saddle-billed stork	Resident	Yes	No	No	No
	*Leptoptilos crumeniferus*	Marabou stork	Resident	No	No	Yes	Yes
	*Mycteria ibis*	Yellow-billed stork	Resident	Yes	No	Yes	Yes
Gruidae	*Balearica regulorum*	Grey crowned crane	Resident	No	No	Yes	No
Hirundinidae	*Hirundo rustica*	Barn swallow	Migrant	No	Yes	No	No
Jacanidae	*Actophilornis africanus*	African Jacana	Resident	Yes	No	No	Yes
Laridae	*Larus cirrocephalus*	Grey-headed gull	Resident	Yes	Yes	Yes	Yes
	*Larus fuscus*	Lesser black-backed gull	Migrant	Yes	No	Yes	Yes
	*Larus ridibundus*	Black-headed gull	Migrant	Yes	No	No	No
Motacillidae	*Motacilla flava*	Yellow wagtail	Migrant	No	Yes	No	No
Pelecanidae	*Pelecanus onocrotalus*	Great white pelican	Migrant	No	No	Yes	Yes
	*Pelecanus rufescens*	Pink-backed pelican	Resident	No	Yes	Yes	Yes
Phalacrocoracidae	*Phalacrocorax africanus*	Long-tailed cormorant	Resident	Yes	Yes	Yes	No
	*Phalacrocorax carbo*	Greater cormorant	Resident	Yes	Yes	Yes	No
Phoenicopterdae	*Phoenicopterus minor*	Lesser flamingo	Resident	No	No	No	Yes
	*Phoenicopterus ruber*	Greater flamingo	Resident	No	No	No	Yes
Recurvirostridae	*Himantopus himantopus*	Black-winged stilt	Migrant	Yes	Yes	Yes	Yes
	*Recurvirostra avosetta*	Pied avocet	Resident	No	No	No	Yes
Rynchopidae	*Rynchops flavirostris*	African skimmer	Resident	No	No	Yes	No
Scolopacidae	*Actitis hypoleucos*	Common sandpiper	Migrant	No	Yes	Yes	Yes
	*Calidris ferruginea*	Curlew sandpiper	Migrant	Yes	No	No	No
	*Calidris minutu*	Little stint	Migrant	Yes	No	Yes	Yes
	*Limosa limosa*	Black-tailed Godwit	Migrant	Yes	No	No	No
	*Philomachus pugnax*	Ruff	Migrant	Yes	Yes	No	No
	*Tringa glareola*	Wood sandpiper	Migrant	No	No	Yes	No
	*Tringa stagnatilis*	Marsh sandpiper	Migrant	Yes	No	No	No
Scopidae	*Scopus umbretta*	Hammerkop	Resident	No	No	Yes	No
Sternidae	*Chlidonias hybridus*	Whiskered tern	Migrant	No	Yes	No	No
	*Chlidonias leucopterus*	White-winged tern	Migrant	Yes	Yes	No	No
	*Sterna nilotica*	Gull-billed tern	Migrant	Yes	Yes	Yes	Yes
Threskiornithidae	*Platalea alba*	African spoonbill	Unknown	No	No	Yes	Yes
	*Plegadis falcinellus*	Glossy ibis	Migrant	Yes	Yes	No	No
	*Threskiornis aethiopicus*	Sacred ibis	Resident	No	Yes	Yes	Yes

### *Salmonella* Detection in Bird Feces and NC Water

*Salmonella* was isolated from 17 of 395 pooled bird fecal samples (Table 2). The prevalence was similar across impacted and protected sites. In contrast, *Salmonella* was far more common (57.1%) in NC water. From each positive pooled fecal sample and water sample, an average of 7 (1–12) and 10 (3–16) PCR confirmed *Salmonella* isolates were obtained, respectively. A total of 121 isolates from birds and 166 from NC water were used in subsequent analyses.

**Table 2:** Prevalence of *Salmonella* in Wild Birds and Environmental Water

Sampling sites	Specimen	No. of positive	Total	% prevalence and 95% CI	% min prevalence^a^	% median prevalence^b^
Queen Elisabeth National Park						
Kazinga Channel	Pooled fecal swabs	6	150	4.0 (1.8–8.5)	0.8	2.4
Bwenda Bay	Pooled fecal swabs	3	48	6.3 (2.1–16.8)	1.3	3.8
Lake Victoria Bird Areas						
Lutembe Bay	Pooled fecal swabs	4	108	3.7 (1.4–9.1)	0.7	2.2
Nakiwogo Area	Pooled fecal swabs	4	89	4.5 (1.7–11.0)	0.9	2.7
	Subtotal	17	395	4.3 (2.7–6.8)	0.9	2.6
Kampala						
Nakivubo Channel	Storm/wastewater	16	28	57.1 (39.1–73.5)		

^a^Minimum prevalence assuming 1 out of 5 individual fecal samples in a pool had *Salmonella*.

^b^Median prevalence assuming 3 out of 5 individual fecal samples in a pool had *Salmonella*.

### Serovar Distribution

**Figure 2:**
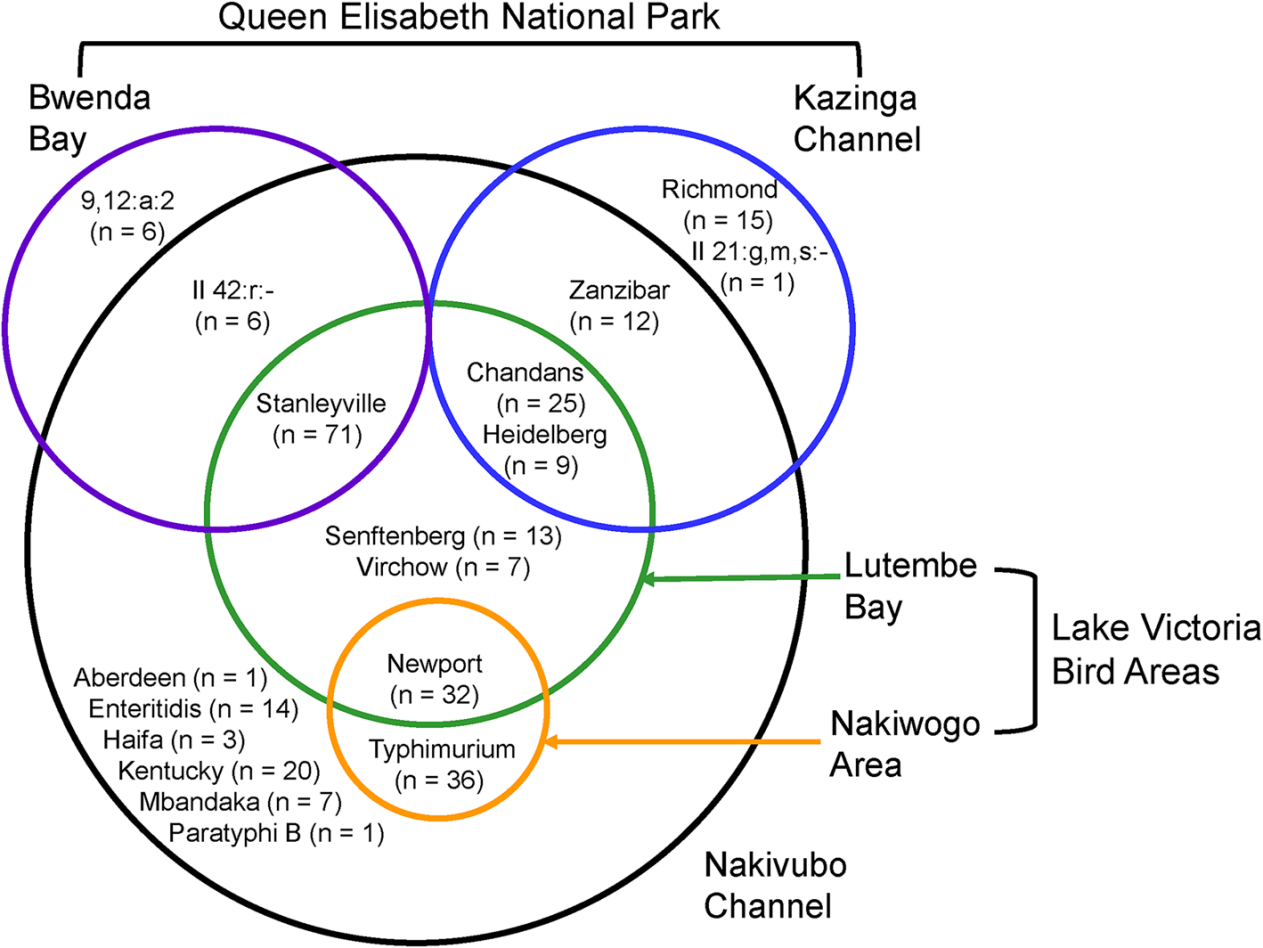
Distribution of *Salmonella* serovars found in wild bird feces and environmental water.

From the 287 *Salmonella* isolates, 18 serovars were identified (Fig. 2). No single serovar was detected across all five sampling sites. *Salmonella* Chandans, *Salmonella* Heidelberg, *Salmonella* Newport, and *Salmonella* Stanleyville were shared across three sampling sites and were always present at Lutembe Bay and NC. *Salmonella* Newport was shared between birds in LVBA and NC water, but not birds at QENP. All serovars found in birds at LVBA also occurred in NC. Serovars unique to sites were also detected.

### Multiple Locus Variable Number Tandem Repeats Analysis

**Figure 3:**
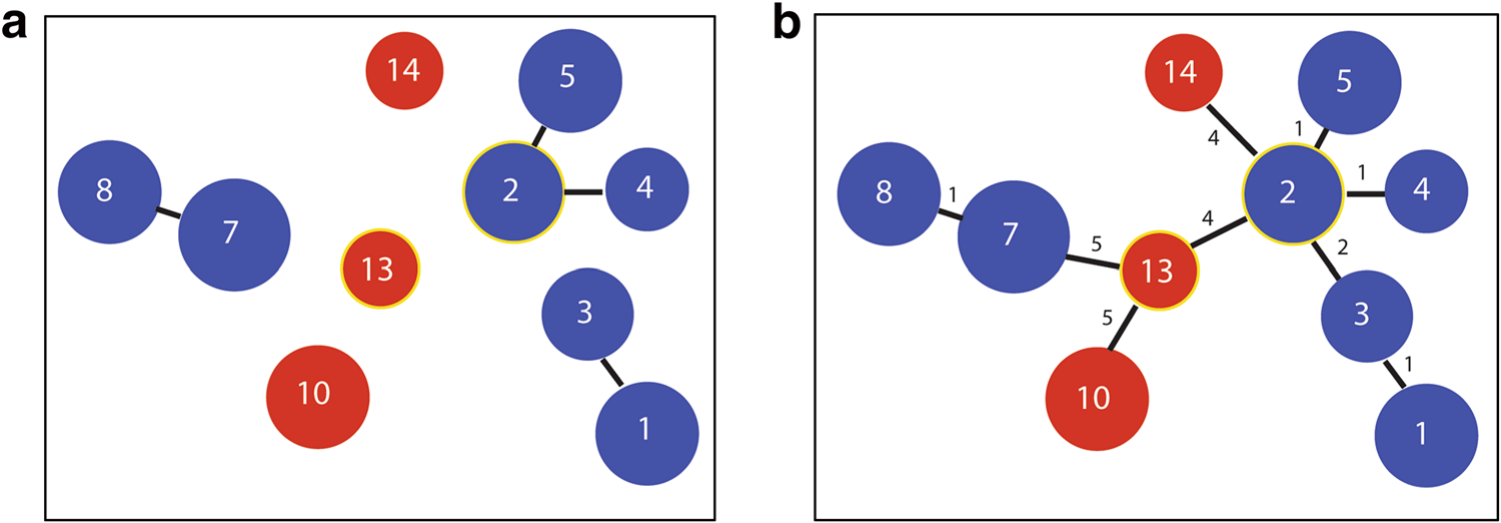
**a**, **b** Minimum spanning tree of *S.* Typhimurium MLVA genotypes constructed using the goeBURST algorithm. There are 10 genotypes represented by blue (birds) or red (NC water) circles. The size of the *circle* is proportional to the number of isolates on a log scale. The *number inside the circle* is the assigned MLVA type. The 7 genotypes from bird isolates cluster into 3 clonal complexes at the single locus variant level (**a**). A full tree with all the genotypes connected and the number of locus variants along the connecting paths is shown in **b**.

We performed MLVA on 29 *S.* Typhimurium isolates from birds at Nakiwogo and 7 isolates from NC water and detected 10 genotypes. A minimum spanning tree of the genotypes was constructed using the goeBURST algorithm to observe genotypic relationships. At the single locus variant level, the genotypes from birds clustered into 3 clonal complexes, and each complex was composed of isolates from a pooled sample (Fig. 3a). With a full goeBURST algorithm, two bird-associated complexes from samples collected within 3 months clustered together and were distinct from another bird associated complex from a sample collected about 9 months earlier (Fig. 3b). The genotypes from NC differed from each other and the bird genotypes by at least 4 alleles.

### Pulsed Field Gel Electrophoresis

We performed PFGE on selected isolates from 8 serovars recovered from at least 2 sampling sites (Table 3). When we compared PFGE patterns of serovars shared between bird sampling sites, there was little evidence of shared PFGE types across sites. For instance, *S.* Chandans and *S*. Heidelberg were shared by birds at Lutembe Bay and Kazinga Channel but had distinct PFGE patterns. Similarly, *S*. Stanleyville was common to birds at Lutembe Bay and Bwenda Bay but had distinct PFGE patterns. Nonetheless, *S*. Newport with similar PFGE types was observed in birds at Lutembe Bay and Nakiwogo.

**Table 3:** PFGE Types in *Salmonella* serovars Common to Birds and Nakivubo Channel

Serovar	PFGE type	Kampala	LVBA		QENP	
		NC	Lutembe	Nakiwogo	Bwenda	Kazinga
Chandans	A	0	0			2
	B	2	3			0
Heidelberg	C^†^	2	0			0
	D^†^	0	0			3
	E	0	2			0
Newport	F*	0	3	0		
	G*	2	0	2		
	H	4	0	0		
II 42:r:-	I	2			1	
Senftenberg	J	2	1			
	K	6	0			
	L	1	0			
Stanleyville	M	3	0		0	
	N^‡^	6	6		0	
	O^‡^	0	3		0	
	P	9	0		2	
	Q	3	0		0	
	R	0	1		0	
Virchow	S	3	0			
	U	0	1			
Zanzibar	V	3				1

Shared superscripts for PFGE types within serovar have Dice similarity index of at least 90%.

*LVBA* Lake Victoria Bird Areas, *QENP* Queen Elisabeth National Park, *NC* Nakivubo Channel.

When we compared PFGE patterns in serovars common to birds and NC, interestingly, similar PFGE types were shared by birds at each site and NC. For example, *S.* Chandans, *S.* Senftenberg, and *S.* Stanleyville with indistinguishable PFGE profiles (B, J, and N, respectively) were common to Lutembe Bay birds and NC. Also, *S*. Newport with similar PFGE types was recovered from NC, and birds at Lutembe Bay and Nakiwogo. Although *S*. Newport, *S*. Senftenberg, and *S*. Stanleyville with similar PFGE types were found in Lutembe Bay birds and NC, other PFGE types were exclusive to NC (H, K, L, and Q). Furthermore, although *S.* Heidelberg was common to Lutembe Bay birds and NC, distinct PFGE patterns were observed in birds (E) and NC (C).

We had expected to observe PFGE differences between QENP birds and NC, but shared serovars between NC and Bwenda Bay (*S.* II 42:r:- and *S*. Stanleyville), and NC and Kazinga Channel (*S.* Heidelberg and *S.* Zanzibar) had similar PFGE patterns. The only shared serovar with PFGE pattern unique to protected area birds was *S*. Chandans.

### Antibiotic Resistance

The *Salmonella* recovered from birds were either susceptible to all 15 antibiotics (pan-susceptible) or had single resistance to sulfisoxazole, except one *S.* Virchow isolate from Lutembe Bay with a multi-drug-resistant profile (Table 4). In NC, 53% of the isolates were pan-susceptible, 19% were resistant to sulfisoxazole, and the remaining isolates were distributed across 17 profiles including multi-drug-resistant types. The multi-drug-resistant profiles were mostly associated with *Salmonella* Kentucky with streptomycin, sulfisoxazole, tetracycline, ciprofloxacin, and nalidixic acid resistance profile.

**Table 4:** Antibiotic Resistance Profiles in *Salmonella* from Birds and Nakivubo Channel

Resistance profile^a^	Kampala	LVBA		QENP		Total
	NC	Lutembe	Nakiwogo	Kazinga	Bwenda	
A	1	0	0	0	0	1
ACSSuSx	6	0	0	0	0	6
ANal	1	0	0	0	0	1
ASuSxT	2	0	0	0	0	2
CSSuCipNal	1	0	0	0	0	1
CSuSxTNal	0	1^a^	0	0	0	1
CipNal	1	0	0	0	0	1
Fox	1	0	0	0	0	1
Gen	1	0	0	0	0	1
Nal	4	0	0	0	0	4
Pansusceptible	88	25	26	22	0	161
SSuT	1	0	0	0	0	1
SSuTCip	1	0	0	0	0	1
SSuTCipNal	17	0	0	0	0	17
Su	32	6	12	10	19	79
SuNal	2	0	0	0	0	2
SuSxT	1	0	0	0	0	1
SuSxTNal	4	0	0	0	0	4
T	2	0	0	0	0	2
Total	166	31	38	32	19	287

^a^ *A* ampicillin, *C* chloramphenicol, *Cip* ciprofloxacin, *Fox* cefoxitin, *Gen* gentamicin, *Nal* nalidixic acid, *S* streptomycin, *Su* sulfisoxazole, *Sx* trimethoprim/sulfamethoxazole, *T* tetracycline, *LVBA* Lake Victoria Bird Areas, *QENP* Queen Elisabeth National Park, *NC* Nakivubo Channel.

## Discussion

Uganda has a high diversity of resident and migratory birds and several habitats that are crucial for their conservation (NatureUganda 2010). We assessed *Salmonella* shedding in apparently healthy water-birds exposed to habitats that varied in the impact of humans on those habitats. Because of the proximity of LVBA to Kampala city, and the relative remoteness of QENP from urban influence, we hypothesized LVBA birds had higher risk of acquiring and shedding environmental-source *Salmonella* than QENP birds. However, we observed no significant difference in *Salmonella* pooled prevalence between LVBA and QENP.

One explanation is that transmission of fecal parasites (including bacteria) is influenced by intensity of habitat use (Nunn et al. 2011). We sampled between October and February which coincides with the wintering season for Afro-Palearctic migrants. We observed high bird numbers at both QENP and LVBA, and although we were unable to estimate number of birds per unit area, the habitats were in intense use relative to off season when only resident birds exist. Modeling has shown that buildup of pathogens in the environment coupled with increased contact leads to increased prevalence in a population during seasonal sharing of resources (Nunn et al. 2014). *Salmonella* prevalence being similar across sampled sites may be directly related to high bird densities and species richness. A way to evaluate this is to determine prevalence when migrants are away. It is possible that when only residents exist, there is less buildup of fecal material, less density dependent transmission, and the environment can be ‘cleaned’ by pathogen decay.

Another explanation for the similar prevalence between sites is that although QENP has less human impact, there is still risk of introducing pathogens from fishing and pastoral communities within and bordering the park. A modeling study showed that continuous introduction of fecal-associated pathogens at the edge of a protected area led to spread within the entire population in the reserve (Nunn et al. 2014). A third explanation is that migrant birds are the source of the *Salmonella* we observed. Alternatively, birds in QENP share habitat with other wild animals, and *Salmonella* could be transmitted between different species.

Our measure of non-typhoidal *Salmonella* prevalence in birds was based on pooled samples. While pooling samples is more sensitive for detecting *Salmonella* compared to individual samples (Arnold et al. 2009, 2011), prevalence cannot be directly determined and instead lies within a range of values (0.2–1 in our study). Our measure has good internal validity for comparing pooled prevalence across sites but makes comparison to previous studies difficult. Nonetheless, our median prevalence inference is comparable to other studies.

*Salmonella* estimated prevalence in our study was greater compared to that in Afro-Palearctic migrants captured at Ottenby bird observatory during fall migration from continental Europe to West Africa (Hernandez et al. 2003) or birds in Greater Chicago (Hamer et al. 2012). In contrast, our prevalence is lower than reports of 15.9% (6.4–43.5%) fecal shedding in migrating cranes at Izumi plain in Japan (Kitadai et al. 2010). A possible explanation is that prevalence is influenced by feeding ecology. Birds in the Ottenby and Chicago studies were passerines where contact with fecal material is less likely, whereas ground roosting water-birds we observed come into contact with feces on the ground or in water, and are more likely to become infected in case of environmental contamination. This is supported by a survey of *Campylobacter* shedding in migratory birds at Ottenby, where higher prevalence occurred in birds that foraged on invertebrates on shorelines, opportunistic feeders, gregarious, and long-lived birds (Waldenstrom et al. 2002).

This study detected 18 different serovars across study sites and found a diverse array of bird associated serovars (*n* = 12) compared to previous studies that mainly found *S.* Typhimurium (Hughes et al. 2008; Kitadai et al. 2010). While prevalence in birds across sampling sites was not significantly different, the serovar distributions were different, and no serovars were shared between all sampling sites. There was similarity between serovars found in water from NC (which contains runoff from Kampala’s urban environment and human source wastewater) and spatially associated birds in LVBA. These sites shared 6 serovars of which 4 had similar PFGE patterns. Paradoxically, a similar pattern of serovar sharing was observed for QENP birds and NC with 5 shared serovars and 4 sharing PFGE patterns. These findings suggest birds across sites were exposed to and likely acquired strains from a human impacted environment. Interestingly, there were 3 serovars shared by QENP and LVBA birds, but none shared PFGE patterns, possibly due to different environments or bird species.

Environmental samples were not collected from QENP because there was no point source reflecting human impact equivalent to NC. While QENP habitats, including water, were assumed to be relatively pristine compared to Lake Victoria region, there are human-associated non-point source impacts because of fishing villages within the park. The sharing of serovars between NC and QENP birds may suggest that birds at QENP are exposed to human-source *Salmonella*. Alternatively, birds that are attracted to human waste may introduce *Salmonella* to bird areas. For instance, Black-winged stilts were observed at all sites on most sampling occasions, and at human waste stabilization ponds.

*Salmonella* Typhimurium was only detected in NC water and birds at Nakiwogo. Although it is cited as the most commonly recovered serovar (Reed et al. 2003; Hughes et al. 2008, 2010), it represented only 8.3% of the serovars we recovered. The genotypes in birds were distinct from those in NC, and most bird isolates were pan-susceptible whereas those from NC were multi-drug resistant. Despite the known wide host range of *S*. Typhimurium, our findings, consistent with other studies, corroborate the conclusion that bird strains are host adapted (Hughes et al. 2008, 2010).

*Salmonella* Stanleyville was common in our study shared between NC, Lutembe Bay (LVBA), and Bwenda Bay (QENP). In a related study, we found similar strains of *S.* Stanleyville in wastewater from a livestock slaughterhouse and human effluent from a wastewater treatment plant (Afema et al. 2016). These facilities discharge wastewater into NC and eventually into Lake Victoria. Finding similar strains of *S.* Stanleyville in Lutembe Bay birds and NC water suggests birds could have acquired them through environmental exposure. Finding similar strains of *S.* Stanleyville in NC water and birds in Bwenda Bay suggests birds that utilize protected and impacted areas could have been exposed to pathogens in urban environments. Alternatively, although human impact in protected areas may be low, human fecal pathogens may still circulate in the environment.

*Salmonella* Chandans was observed in birds from both conserved and impacted areas, but this serovar is rare in humans and livestock (Madden et al. 2007). As expected, identical PFGE and antibiotic resistance patterns occurred in LVBA and NC but were distinct from those in QENP.

This study used a noninvasive sampling technique which still provides adequate initial insights into *Salmonella* ecology in birds. However capturing and obtaining samples from known species would have provided information on risk factors associated with *Salmonella* shedding in different guilds and age groups. Also, sampling was limited to October–March when migrants occur at these sites. Sampling throughout the year would have provided information on *Salmonella* occurrence during the non-migratory season. In addition, we used PFGE and MLVA to establish genotypic relationships; however, phylogenetic analysis of single-nucleotide polymorphisms based on whole genome sequence data would have provided better resolution of strain variations.

Future collaborations with ornithologists to capture birds would be invaluable to obtain health and biological data for monitoring zoonotic pathogens. Monitoring *Salmonella* in lake water and soils on roosting grounds would be invaluable in understanding *Salmonella* ecology. Furthermore, investigating *Salmonella* strains in humans residing in fishing and urban communities would provide insights into transmission processes at these human–wildlife interfaces. Satellite tracking and microbiological assessments would help in understanding the spatial extent of pathogen spread by birds.

## Conclusions

Salmonellosis continues to exert a huge burden on public health worldwide (Majowicz et al. 2010) and epidemics with mortalities have occurred in birds (Velarde et al. 2012; Giovannini et al. 2013). We have shown that *Salmonella* is shed by birds in both conserved and human-impacted areas, more strains exist in the urban environment than in birds at all sites, some strains are adapted to birds, and that several strains in birds are indistinguishable from strains in the environment. Our results indicate that there is risk of different strains disseminating between the urban environment and birds across sites. Furthermore, *Salmonella* could reemerge particularly in birds at LVBA because diverse species of resident and migratory birds congregate at high densities and the spatial connection with Kampala city exposes birds to human source pathogens. While the significance of *Salmonella* shedding on bird health is unknown, it may affect population vitality during stressful periods or when coinfections occur (Klaassen et al. 2012). It is also possible that birds could spread strains acquired from impacted environments to humans, other species and areas and this calls for additional studies.
